# *Eucalyptus grandis* WRKY genes provide insight into the role of arbuscular mycorrhizal symbiosis in defense against *Ralstonia solanacearum*

**DOI:** 10.3389/fpls.2025.1510196

**Published:** 2025-02-07

**Authors:** Jianlang Zhang, Xinzhu Yang, Chunyu Huo, Xinyi Fan, Qiutong Liu, Zhihong Liu, Yu Su, Zujing Chen

**Affiliations:** ^1^ College of Forestry and Landscape Architecture, South China Agricultural University, Guangzhou, China; ^2^ Guangzhou Collaborative Innovation Center on Science-tech of Ecology and Landscape, Guangzhou Institute of Forestry and Landscape Architect, Guangzhou, China; ^3^ State Key Laboratory of Conservation and Utilization of Subtropical Agro-Bioresources, Guangdong Laboratory for Lingnan Modern Agriculture, South China Agricultural University, Guangzhou, China

**Keywords:** WRKY, arbuscular mycorrhizal fungi (AMFs), *Rhizophagus irregularis*, *Eucalyptus grandis*, *Ralstonia solanacearum*, plant-AMF-bacterium interaction

## Abstract

**Introduction:**

WRKY transcription factors are essential for plant growth, health, and responses to biotic and abiotic stress.

**Methods:**

In this study, we performed a deep *in silico* characterization of the WRKY gene family in the genome of *Eucalyptus grandis*. We also analyzed the expression profiles of these genes upon colonization by the arbuscular mycorrhizal fungus (AMF) *Rhizophagus irregularis* (*Ri*) and infection with the bacterial pathogen *Ralstonia solanacearum* (*Rs*).

**Results:**

A total of 117 *EgWRKYs* were identified. Phylogenetic analysis divided the EgWRKY proteins into three groups: group I (21 proteins, 17.95%), group II (65 proteins, 55.56%), and group III (24 proteins, 20.51%). Additionally, seven EgWRKY proteins (5.98%) were categorized into group IV due to the absence of the WRKY domain or zinc-finger structure. All *EgWRKY* genes are distributed irregularly across the 11 chromosomes, with 25 pairs identified as segmental duplicates and four as tandem duplicates. The promoter regions of 50% of members of each subfamily contain plant hormone-related cis-elements associated with defense responses, such as ABREs, TGACG motifs, and CGTCA motifs. All subfamilies (except for group IV-b and IV-c) contain AW-boxes, which are related to mycorrhizal induction. Furthermore, transcriptomic analysis revealed that 21 *EgWRKYs* were responsive to the AMF *Ri*, with 13 and 8 genes strongly up- and downregulated, respectively. Several genes (including *EgWRKY116, EgWRKY62*, and *EgWRKY107*) were significantly induced by *Ri*; these genes might enhance the defense of *E. grandis* against *Rs*.

**Discussion:**

Therefore, we identified *E. grandis* WRKY genes that are regulated by AMF colonization, some of which might improve the defense of *E. grandis* against *R. solanacearum*. These findings provide insights into *E. grandis* WRKY genes involved in interactions among the host plant, AMFs, and *R. solanacearum*.

## 1 Introduction

*Eucalyptus grandis*, a globally planted tree species, is known for its exceptional growth rate and diverse characteristics (Mao et al., 2024). This plant is highly productive, adaptable, versatile, and valuable (Yu et al., 2019; Mao et al., 2024). However, *Eucalyptus* trees are susceptible to a wide range of pathogens, which lead to substantial losses (Yang et al., 2022; Ahmad et al., 2024). More than 80% of *Eucalyptus* species are prone to disease. In particular, the incidence of bacterial wilt ranges from 60.6% to 72.4%, leading to severe economic damage to *Eucalyptus* nurseries (Ferreira et al., 2018).

Bacterial wilt disease, one of the most destructive plant diseases worldwide, is caused by the soil-born pathogen *Ralstonia solanacearum* (*Rs*). This disease poses a major constraint to the production of more than 250 plants worldwide, including forest trees, such as *Eucalyptus* species (Ferreira et al., 2018; Vailleau and Genin, 2023). *Rs* can survive for extended periods under natural conditions (Grey and Steck, 2001). *Rs* penetrates plants through wounds, root tips, and secondary root emergence spots and grows and multiplies within the intercellular gelatinous layer of the plant. This process leads to plasmolysis of epidermal cells, forming cavities, and colonization within the root cortex (Monther et al., 2012; Xue et al., 2020). *Rs* further invades the xylem ducts from plant epidermal cells. The growth cycle of this pathogen within vascular bundles is regulated by the quorum sensing system (Li et al., 2023a). This system mediates the transition of *Rs* from the early stages of rapid division to later stages of slow growth, where the pathogen uses fewer nutrients and produces and disperses virulence factors (Lowe-Power et al., 2018). *Rs* invasion severely impairs water transport and nutrient metabolism in the plant, ultimately leading to the blockage of the water transport system (Yu et al., 2024). Consequently, plants affected by bacterial wilt disease show withering and premature death (Yu et al., 2024). Since chemical measures are not effective in protecting plants from this disease, biological control methods might represent a feasible approach for *Rs* control in the future (Yuliar et al., 2015; Ferreira et al., 2018).

Arbuscular mycorrhizal fungi (AMFs) are plant symbionts with beneficial effects on plant growth and health, as they provide plants with nutrients and induce host resistance to pathogens (Fiorilli et al., 2024; Duan et al., 2024; Martin and van der Heijden, 2024). On one hand, AMFs occupy the ecological niche of pathogenic bacteria, altering the microbial composition in the rhizosphere and promoting the growth of beneficial microorganisms, such as *Pseudomonas fluorescens* (Li et al., 2023b). This improves the antagonistic effects of the soil against pathogenic bacteria (Lahlali et al., 2022; Basiru et al., 2023). On the other hand, AMFs induce host plant defense responses, such as mycorrhizal-induced resistance and the mitogen-activated protein kinase (MAPK) cascade, to enhance plant stress tolerance (Cameron et al., 2013; Tian et al., 2021). Furthermore, AMFs trigger a robust and rapid immune response in the host plant, improving plant disease resistance by activating defense genes, including WRKY transcription factors (TFs) (Gallou et al., 2012).

WRKY TFs are a widely studied class of plant defense-responsive proteins that regulate stress resistance and plant-specific growth and development (Yan et al., 2022; Javed and Gao, 2023; Wang et al., 2023). *SlWRKY75* in tomato (*Solanum lycopersicum*) positively regulates the plant defense response against *Pseudomonas syringae* by maintaining the homeostasis of plant growth hormones. *SlWRKY75* directly activates the expression of *SlGH3.3* by binding to the W-box element in its promoter (Yang et al., 2024). In chili pepper (*Capsicum annuum*), silencing *CaWRKY22*, *CaWRKY27*, or *CaWRKY40* enhanced plant susceptibility to *R. solanacearum* (Dang et al., 2013, 2014; Hussain et al., 2018); *CaWRKY27b*, *CaWRKY40*, and *CaWRKY22* might form a module that regulates plant tolerance to high temperatures and high humidity as well as resistance to *R. solanacearum* (Hussain et al., 2018; Yang et al., 2022). WRKY TFs also play important roles in plant–AMF**–**bacterium interactions (Wang et al., 2022; Silvestri et al., 2024). *MdWRKY40* provides protection against *Fusarium solani* infection in mycorrhizal apple (*Malus domestica* Borkh) seedlings (Wang et al., 2022). Furthermore, *MtWRKY69* is associated with AMF colonization levels in *Medicago truncatula* (Silvestri et al., 2024).

WRKY proteins possess a conserved WRKY motif (WRKYGQK) and a zinc-finger structure following the C_2_H_2_, C_2_HC motif (Rushton et al., 2010; Yan et al., 2022). WRKY proteins can be categorized into four groups, I–IV. Group I members possess two WRKY motifs and one C_2_H_2_-type zinc finger. Group II and group III members possess a single WRKY motif. However, group II members are characterized by a C_2_H_2_-type zinc finger, while group III members possess a C_2_HC-type zinc finger. Group II WRKYs are further classified into five subgroups (IIa–IIe) based on the sequences of their DNA-binding domains and the structures of their zinc fingers, while group III is divided into subgroups III-a and III-b. Group IV members have incomplete or partial WRKY structural domains and do not possess zinc-finger motifs, suggesting that these proteins may have lost their roles as WRKY transcription factors (Zhang and Wang, 2005; Chen et al., 2019; Javed et al., 2022; Yan et al., 2022). Analyzing *EgWRKY* genes for their potential induction by AMF to enhance disease resistance could open new possibilities for *Eucalyptus* disease control and breeding programs.

We previously demonstrated that inoculating *E. grandis* with the AMF *Funneliformis mosseae* or *Rhizophagus irregularis* drastically increased the activities of defense-related enzymes and enhanced the defense of *E. grandis* against *R. solanacearum* (Huang et al., 2023). In this study, we performed a genome-wide analysis of WRKY genes in *E. grandis*. We used a tripartite system involving *E. grandis–*AMF–*R. solanacearum* to identify *E. grandis* WRKY genes that are induced by AMF and *R. solanacearum*. These genes might play roles in enhancing the defense of *E. grandis* against bacterial pathogens.

## 2 Materials and methods

### 2.1 Identification of WRKY genes in the *Eucalyptus grandis* genome

All *E. grandis* genome sequences and annotation were obtained from the NCBI nucleotide sequence repository (https://www.ncbi.nlm.nih.gov/datasets/taxonomy/71139/). The Pfam database (http://pfam.sanger.ac.uk/) was used to obtain an HMM file of predicted WRKY structural domain (PF03106). A basic HMM search using TBtools v2.086 was conducted to identify WRKY family proteins in the *E. grandis* protein database with an e-value cutoff of 1 × 10^−5^ (Chen et al., 2023). The NCBI CDD (http://www.ncbi.nlm.nih.gov/cdd/) and SMART databases (http://smart.embl.de/) were used to confirm the existence of the WRKY domain in all *Eucalyptus* WRKY proteins.

### 2.2 Phylogenetic analysis and multiple sequence alignment

The Arabidopsis (*Arabidopsis thaliana*) WRKY (AtWRKY) protein sequences were downloaded from Phytozome (https://phytozome-next.jgi.doe.gov/) (Goodstein et al., 2012). Multiple sequence alignment of AtWRKY and EgWRKY proteins was performed using ClustalW with MEGA11.0 software (Tamura et al., 2021) with default parameters and used to construct a maximum likelihood phylogenetic tree of AtWRKY and EgWRKY protein sequences.

### 2.3 Gene structure, chromosomal distribution, and Circos plots

TBtools (Chen et al., 2023) was used to confirm the chromosomal locations and gene structures of the *EgWRKYs* and to generate a chromosome distribution map and gene structures for all *EgWRKYs*. To gain a better understanding of the functions of the *EgWRKYs*, the deduced protein sequences were analyzed using MEME (Multiple Expectation Maximization for Motif Elicitation: http://meme-suite.org/tools/meme/) to identify and examine conserved motifs, with the following parameters: the repeat count was adjusted to zero or one, with a maximum of ten motifs (Bailey et al., 2009). TBtools was also utilized to generate Circos plots. The One Step MCScanX program in TBtools was used to identify and investigate duplication types and collinear blocks and to build Circos plots.

### 2.4 Physiochemical parameters and promoter analysis of *EgWRKYs*

The isoelectric point (pI) and molecular weight (Mw) functions in ExPASy (https://web.expasy.org/protparam/) were used to estimate the pI and Mw of full-length EgWRKY proteins (Gasteiger et al., 2003). TBtools was used to harvest 2,000 base-pair (bp) sequences upstream of the start codon (ATG) of each *EgWRKY* gene in the *Eucalyptus* genome (Chen et al., 2023). After analyzing sequences with PlantCARE online (Lescot et al., 2002) and New PLACE (https://www.dna.affrc.go.jp/PLACE/?action=newplace), *cis*-acting elements were identified and their distribution in each promoter was determined using TBtools.

### 2.5 Construction of an *E. grandis*–AMF–*R. solanacearum* interaction system

*E. grandis* seeds were surface sterilized with 1% NaClO for 15 min and placed on sterilized quartz sand for germination. After 14 days, seedlings with uniform growth status were transferred to individual pots and inoculated with *R. irregularis* 197198 (*Ri*) at the roots using 400 spores per plant. At 45 days post inoculation (dpi) with *Ri*, mycorrhizal colonization was detected in *E. grandis* roots after WGA488 staining and observed under a fluorescence microscope (Trouvelot et al., 1986). At 90 dpi, the roots were watered with 20 mL of a 1 × 10^9^ CFU/mL suspension of *R. solanacearum*. The plants were placed in an artificial climatic chamber with 16 h of light, 30°C, 80% relative humidity, light intensity of 100–200 Wm^–2^, and 8 h of darkness at 28°C, 80% relative humidity. Mycorrhizal and non-mycorrhizal *E. grandis* root samples were collected at 0, 24, 48, and 96 hours post-infection (hpi) with *Rs*, flash-frozen in liquid nitrogen, and stored at –80°C.

### 2.6 Gene expression analysis

Total RNA was extracted from the samples using a Plant RNA Kit (BIO-TEK R6827, OMEGA). The integrity of the total RNA was assessed using 1.5% agarose gel electrophoresis, while its concentration and quality were evaluated using an ultra microspectrophotometer (Nanophotometer N50 Touch, Implem). RNAs from the roots of non-mycorrhizal and mycorrhizal *E. grandis* seedlings infected with *Rs* at 0, 24, 48, and 96 hpi were sent to Lianchuan Biotechnology, Hangzhou for cDNA library construction and double-end sequencing of the cDNA library using the Illumina NovaSeq™ 6000 platform (LC-Bio Technologies Co., Ltd., Hangzhou, China). The RNA-Seq (PRJNA893422) data were annotated based on the *E. grandis* reference genome. The ballgown package was used for segmental per kilobase million (FPKM) quantification. FPKM = all exon sequences/matched sequences × exon length (KB). At the same time, TBtools was used to perform normalization analysis of the FPKM data and to identify significant differences (|log2FC ≥ 1| was considered to be significant). The heatmap diagram was also constructed with log_2_ values using TBtools.

## 3 Results

### 3.1 Identification of *EgWRKYs* in the *Eucalyptus grandis* genome

**Figure 1:**
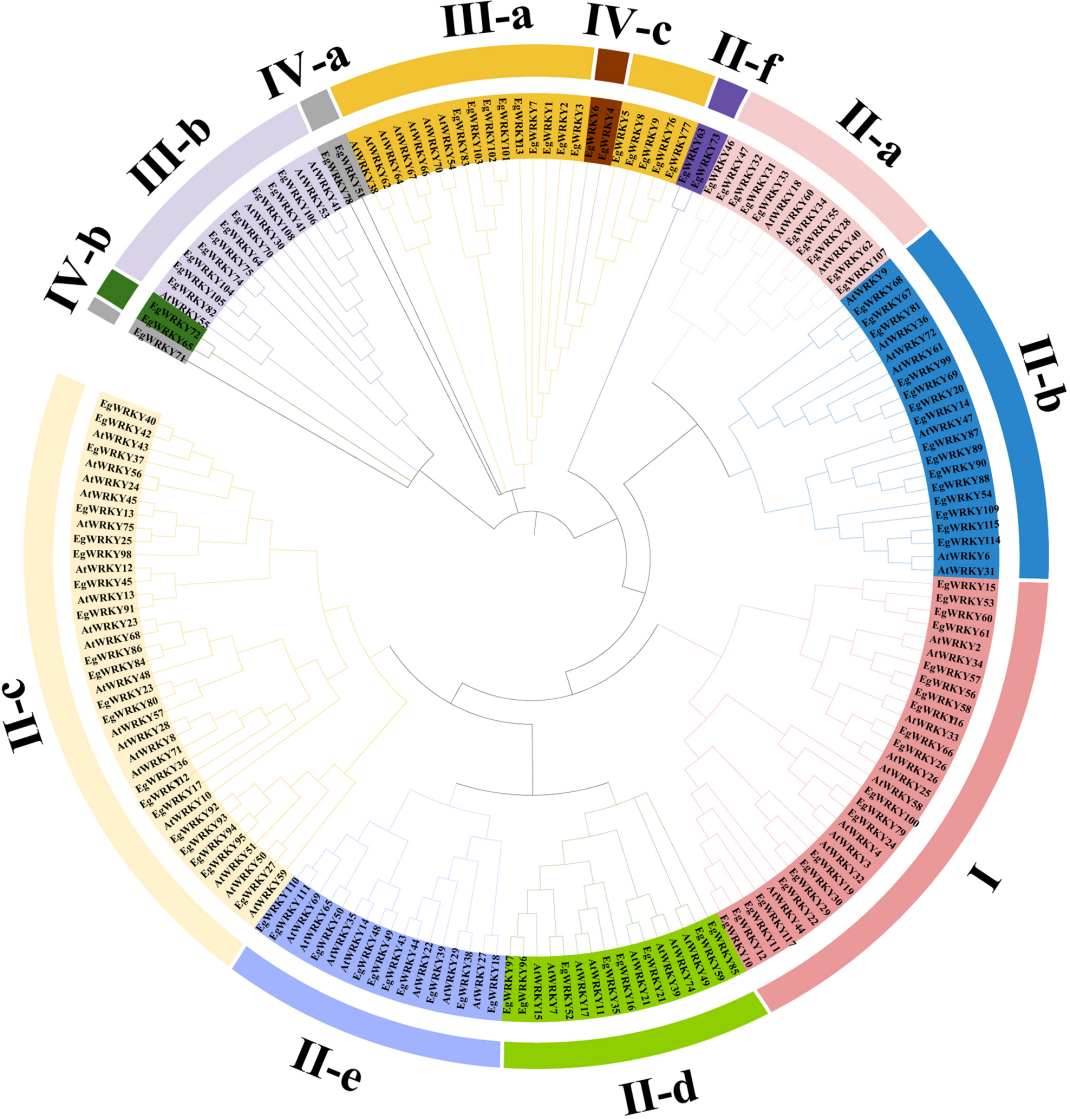
Phylogenetic tree of WRKY proteins in *E. grandis* and *A. thaliana*. The tree was constructed with MEGA11.0 using the Maximal likelihood (ML) method following ClustalW alignment of all full-length protein sequences. Different colors denote group I, group II-a–II-f, group III-a–III-b, and group IV-a–IV-c.

We identified 117 WRKY sequences in the *E. grandis* genome (**Figure 1**, **Supplementary Table S1**). The *EgWRKYs* encode proteins ranging from 139 (EgWRKY7) to 1,831 (EgWRKY63) amino acids long. The molecular weights of the EgWRKYs range from 15.796 kDa (EgWRKY72) to 204.607 kDa (EgWRKY63), with the majority falling between 20 and 85 kDa. The calculated isoelectric points (pI) range from 4.75 (EgWRKY85) to 11 (EgWRKY80), with an average of 7.11. The pI values of 48 EgWRKYs are greater than 7, while the pI values of 69 EgWRKYs are less than 7.

### 3.2 Phylogenetic and multiple sequence alignment of the *EgWRKY* gene family

**Figure 2:**
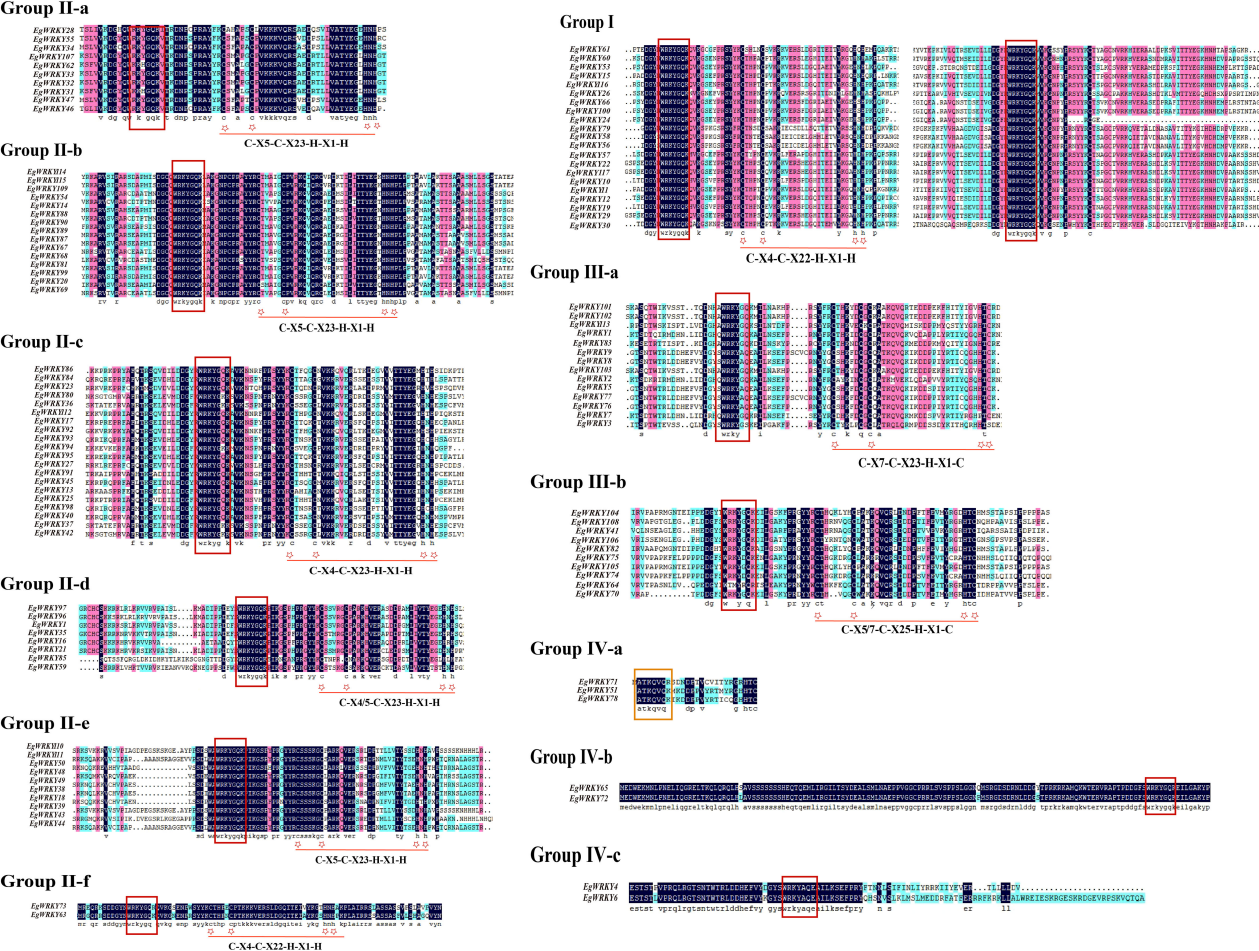
Multiple sequence alignment of the conserved domains of EgWRKY proteins from *E. grandis*. Black shading indicates identical amino acid sequences in various WRKYs. The zinc-finger structure is denoted by an asterisk and is underlined, while the WRKY motif is denoted by a red box.

Sequence analysis revealed a highly conserved middle WRKY domain (WD) in the EgWRKY proteins (**Figure 2**), covering approximately 60 amino acid residues. The WRKY proteins were classified into four types based on the specific zinc-finger motifs and the number of WDs. Group I members possess two WRKY motifs, while group II and III members possess a single WRKY motif (**Figure 1**). Group I and II members harbor a C_2_H_2_ zinc-finger motif following the WRKY structural domain, whereas group III WRKY proteins contain a C_2_HC zinc-finger motif (**Figure 2**). By combining the results of phylogenetic analysis and analysis of zinc-finger structures, we further classified the EgWRKYs into subgroups. Cluster II was classified into six subgroups (II-a–II-f): II-a (C-X_5_-C-X_23_-H-X_1_-H), II-b (C-X_5_-C-X_23_-H-X_1_-H), II-c (C-X_4_-C-X_23_-H-X_1_-H), II-d (C-X_4/5_-C-X_23_-H-X_1_-H), II-e (C-X_5_-C-X_23_-H-X_1_-H), and II-f (C-X_4_-C-X_22_-H-X_1_-H). Group III was subdivided into two subgroups: III-a (C-X_7_-C-X_23_-H-X_1_-C) and IIIb (C-X_5/7_-C-X_25_-H-X_1_-C) (**Figures 1**, **2**). By contrast, the presence of incomplete WRKY domains (KQVQ) or zinc fingers in group IV (**Figure 2**) suggests that EgWRKY65, EgWRKY72, EgWRKY78, EgWRKY51, and EgWRKY71 may have lost WRKY TF function. In addition to variations in the zinc-finger motif, mutations in the WRKYGQK sequence were also observed in *E. grandis*, including WKKHGQK (II-a), WRKYGKK (II-c), WRKYGQR (II-f), WRKYAQE (III-a), WRKYGSK (III-a), WRKYDQK (III-b), and WTMYRQR(III-b). Since EgWRKY3 and EgWRKY7 each contain a zinc-finger motif, they were classified into group III-a rather than group IV, although their zinc fingers exhibit minor differences with those of other group III-a members.

### 3.3 Chromosomal mapping and analysis of the duplication of *EgWRKY* genes

**Figure 3:**
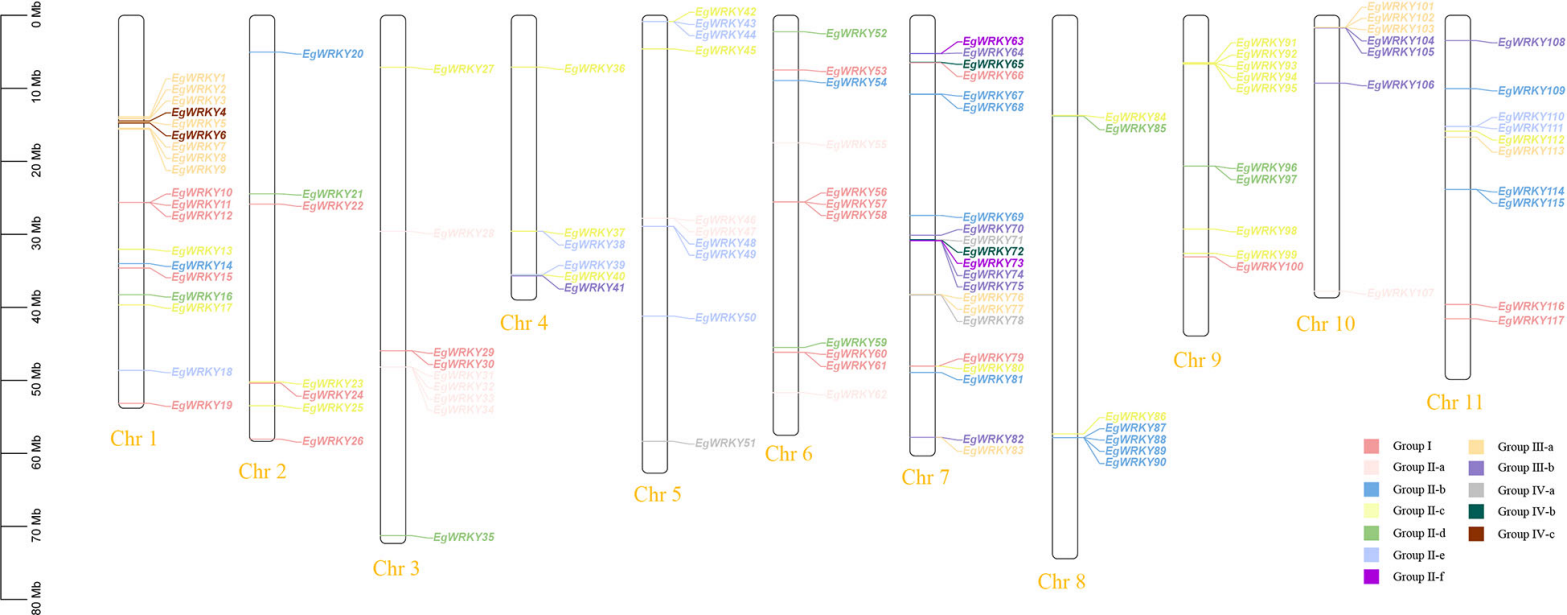
Chromosome mapping of the *EgWRKY*s. The scale represents an 80-Mb chromosomal distance. Groups I, II-a, II-b, II-c, II-d, II-e, II-f, III-a, III-b, IV-a, IV-b, and IV-c are denoted by dark pink, light pink, blue, light green, medium green, cyan, fuchsia, orange, purple, gray, dark green, and brown, respectively.

The 117 *EgWRKYs* are unevenly distributed across all 11 chromosomes of *E. grandis* (**Figure 3**). Most of these *EgWRKY* genes are located on chromosomes 1 and 7, accounting for 15.38% (18 genes) and 23.08% (21 genes) of the total number of genes, respectively. Six *EgWRKY* genes are dispersed on chromosomes 2 and 4, each accounting for 5.12%. Chromosome localization mapping also revealed that *EgWRKYs* exist in multiple gene clusters on different chromosomes, such as *EgWRKY87–EgWRKY90* on chromosome 8 and *EgWRKY91–EgWRKY95* on chromosome 9, with most members of the same cluster categorized in the same subfamilies.

**Figure 4:**
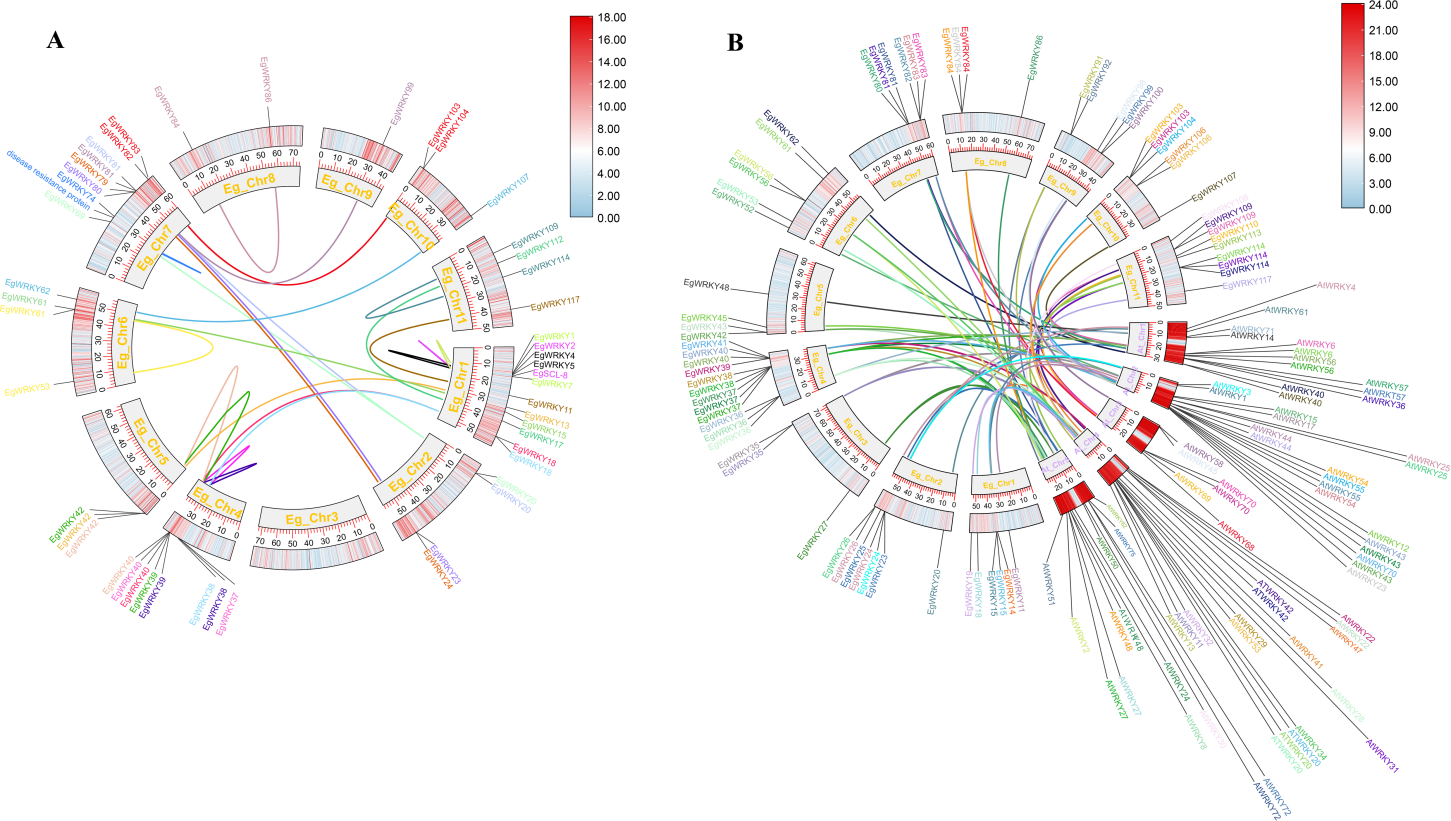
Circos plots of the chromosomal locations of *EgWRKYs* with duplication links. **(A)** Duplication of *EgWRKYs* within the *E. grandis* genome. **(B)** Duplication of *EgWRKY* orthologs within the Arabidopsis genome. A pair of genes with the same color connected by a line represents a duplication relationship. The outer ring shows the density of genes on the chromosome, with redder colors representing greater density.

Collinearity analysis revealed 21 pairs of segmental duplicates and 4 tandem duplicates among the 117 *EgWRKYs*. The four tandem duplicates include *EgWRKY4* with *EgWRKY5*, *EgWRKY2* with *EgSCL8* (encoding scarecrow-like protein 8), *EgWRKY1* with *EgWRKY7*, and *EgWRKY74* with *E. grandis* “Probable Disease Resistance Protein” (XM_039316797.1) (**Figure 4A**, **Supplementary Table S2**). Additionally, we identified 68 pairs of segmental duplicates between *E. grandis* and Arabidopsis WRKYs (**Figure 4B**, **Supplementary Table S3**).

### 3.4 Structural analysis of *EgWRKY* genes

**Figure 5:**
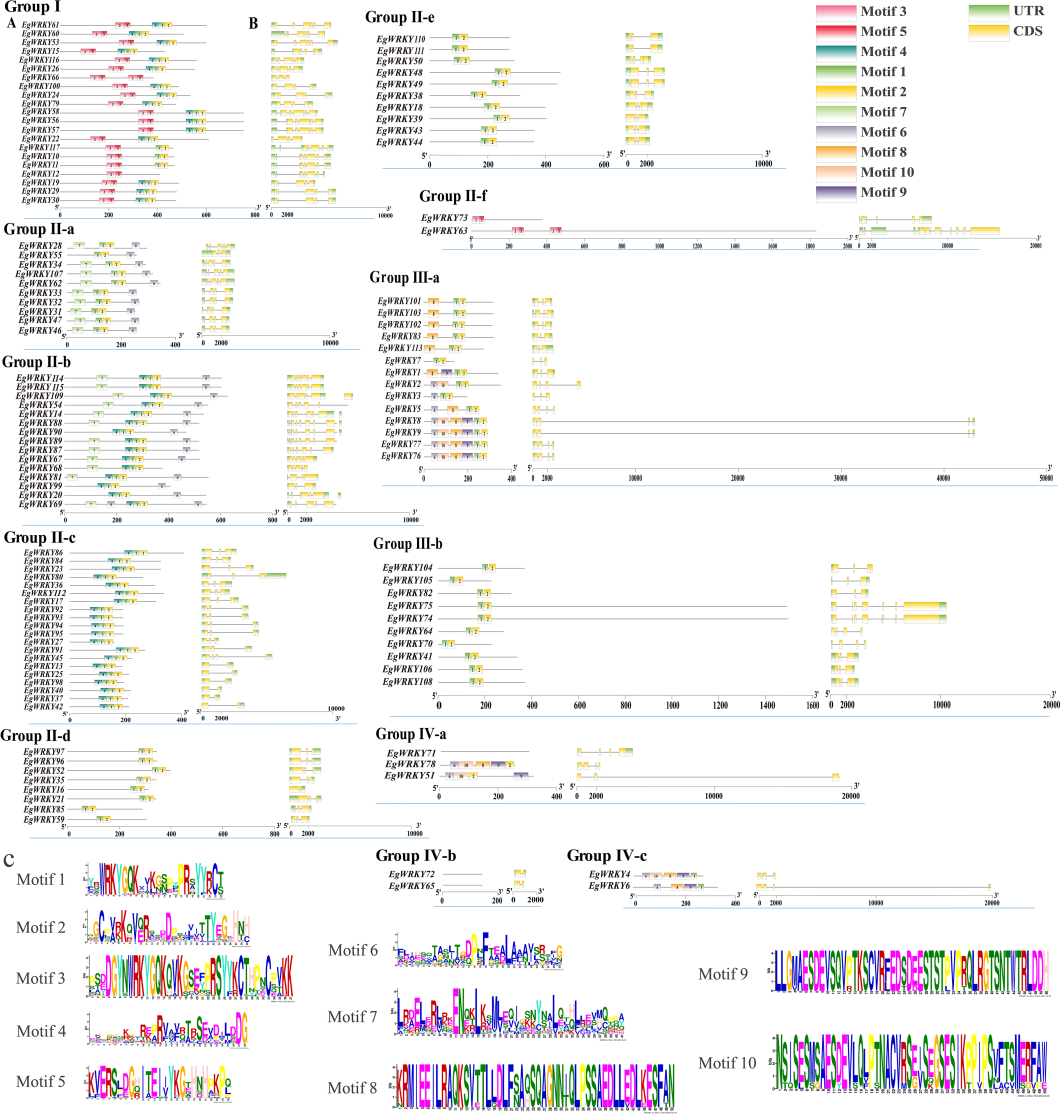
Gene structures and conserved motif analysis of *EgWRKY*s. **(A)** The conserved motifs of the *EgWRKYs*. **(B)** The exon–intron structures of the *EgWRKYs*. UTRs are denoted by green boxes, CDSs by yellow boxes, and introns by narrow black lines. Rectangles of varying colors are used to depict each conserved motif. The length of the box is equivalent to the length of the motif. **(C)** WebLogos of the amino acid composition of each motif.

The quantity of introns and exons differ among the *EgWRKY*s. Specifically, *EgWRKY* family genes contain one to twelve introns and two to nine exons. In terms of exon length and intron number, members of the same subfamily exhibit comparable exon/intron distribution patterns. For example, most members of subfamilies II-c, II-d, and II-e have only one to two introns, while most members of subfamilies I, II-a, and IV possess three or more introns. Notably, subfamily IV members *EgWRKY51* and *EgWRKY6* contain an intron of more than 18,000 bp, and subfamily IIIa members *EgWRKY5* and *EgWRKY9* possess an even larger intron of ~38,000 bp (**Figure 5B**, **Supplementary Table S1**).

Ten motifs were predicted in the *EgWRKYs*, with two to six motifs present in each *EgWRKY* except for some members of group IV (**Figures 5A–C**). Some motifs are shared by most members of groups I–III, such as motif 1 and motif 2, while others are specific to the subfamily. For example, motif 3 and motif 5 are present only in groups I and II-f. However, members of group I contain motif 4, motif 1, and motif 2, whereas group II-f members lack these motifs. This unique structure may cause members of subfamily group II-f to be outliers in the evolutionary tree (**Figures 1**, **5A**). Most group II-a and group II-b members possess motif 7 and motif 6, while group II-a members contain an additional motif 4 linked to motif 1 than group II-b; groups II-d, II-e, and III-b only contain motif 1 and motif 2. In addition, groups III-a, IV-a, and IV-c contain similar motifs in terms of number and arrangement, which could explain why they were grouped in the same branch (**Figure 1**), whereas *EgWRKY4*, *EgWRKY6*, *EgWRKY51*, and *EgWRKY78* were classified in subfamily IV based on the structures of their zinc fingers (**Figure 2**).

### 3.5 *Cis*-elements in the *EgWRKY* promoters

**Figure 6:**
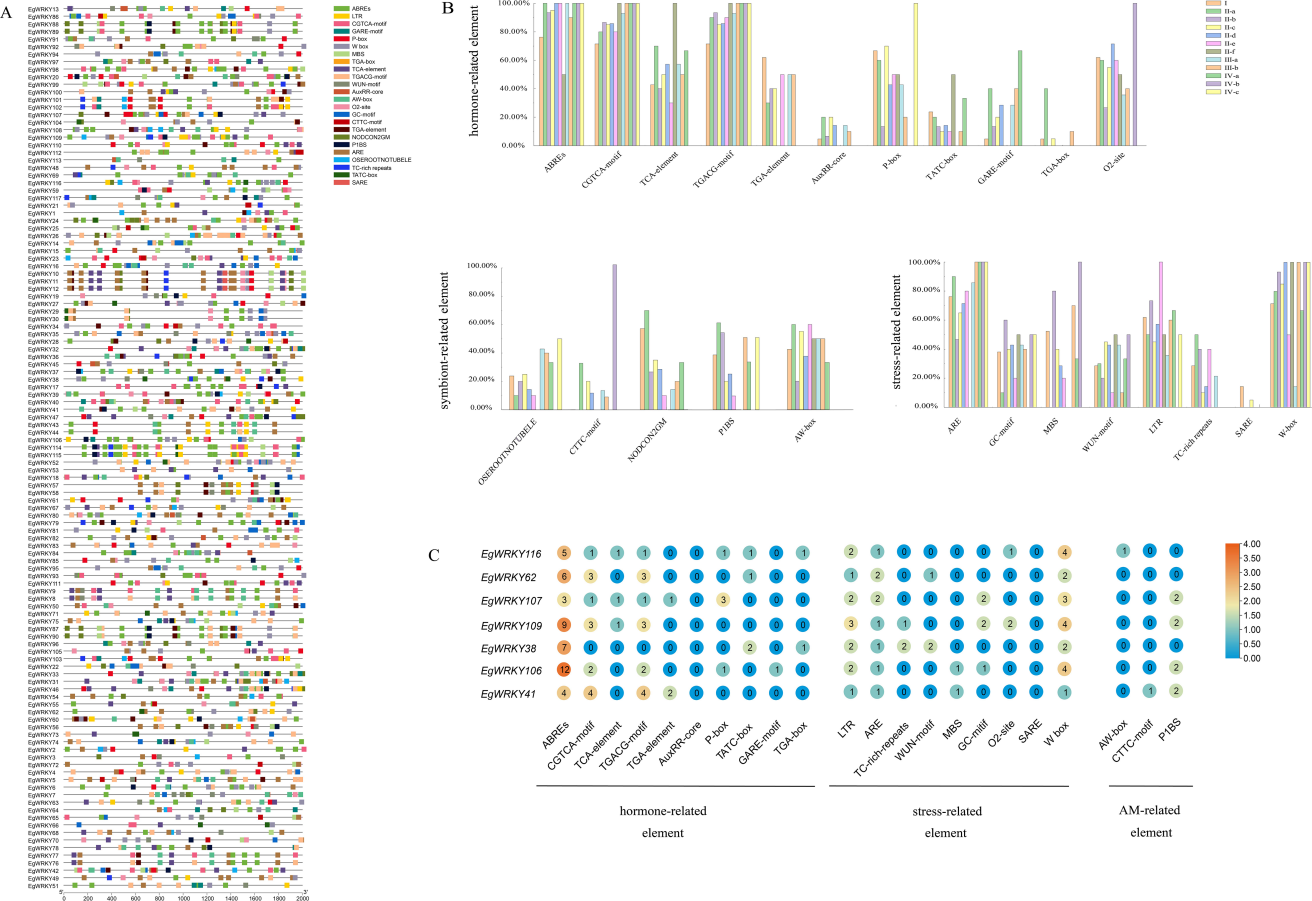
Identification of *cis*-acting elements in the promoter regions of *EgWRKY* genes. **(A)** Each element is depicted by a colored rectangle. The length of the rectangle is equivalent to the length of the element. Different colors correspond to different members of the group. **(B)** Proportional distribution of individual *cis*-acting elements within the promoter regions of *EgWRKYs*. **(C)** Number of *cis*-acting elements in *EgWRKYs* that exhibited a response to *Rs* infection and inoculation with AMFs.

To investigate the putative regulatory mechanisms of *EgWRKY* genes, we identified the *cis*-elements in the 2-kb sequences upstream of their translation start sites (ATGs) and visualized them using TBtools (**Figure 6A**). The *cis*-elements ABREs (abscisic acid responsive), TGACG motifs (MeJA responsive), and CGTCA motifs (MeJA responsive), which are associated with defense hormones, accounted for more than 50% of all *EgWRKY* subfamilies members (**Figure 6B**). Abscisic acid and jasmonic acid are important phytohormones in the spatio-temporal dynamics of AMF-induced resistance (Cameron et al., 2013). These findings suggest that these *EgWRKYs* might be involved in the mycorrhiza-induced resistance of *Eucalyptus* against *Rs*. The presence of auxin-responsive elements, such as TGA-box and AuxRR-core elements (Lescot et al., 2002), suggests that some *EgWRKYs* may play a role in regulating plant growth. Notably, 60% of subgroup II-b members contain GC-motif elements, whereas 80% and 100% of subgroup II-b and IV-b members contain MBS elements, 73% and 100% of subgroup II-b and II-e members contain LTR elements, and 14% and 5% subgroup I and II-c members contain SARE elements, respectively. Finally, whereas subgroup II-f members lack ARE elements, all subgroup III-b, IV-a, IV-b, and IV-c members contain this element.

**Figure 7:**
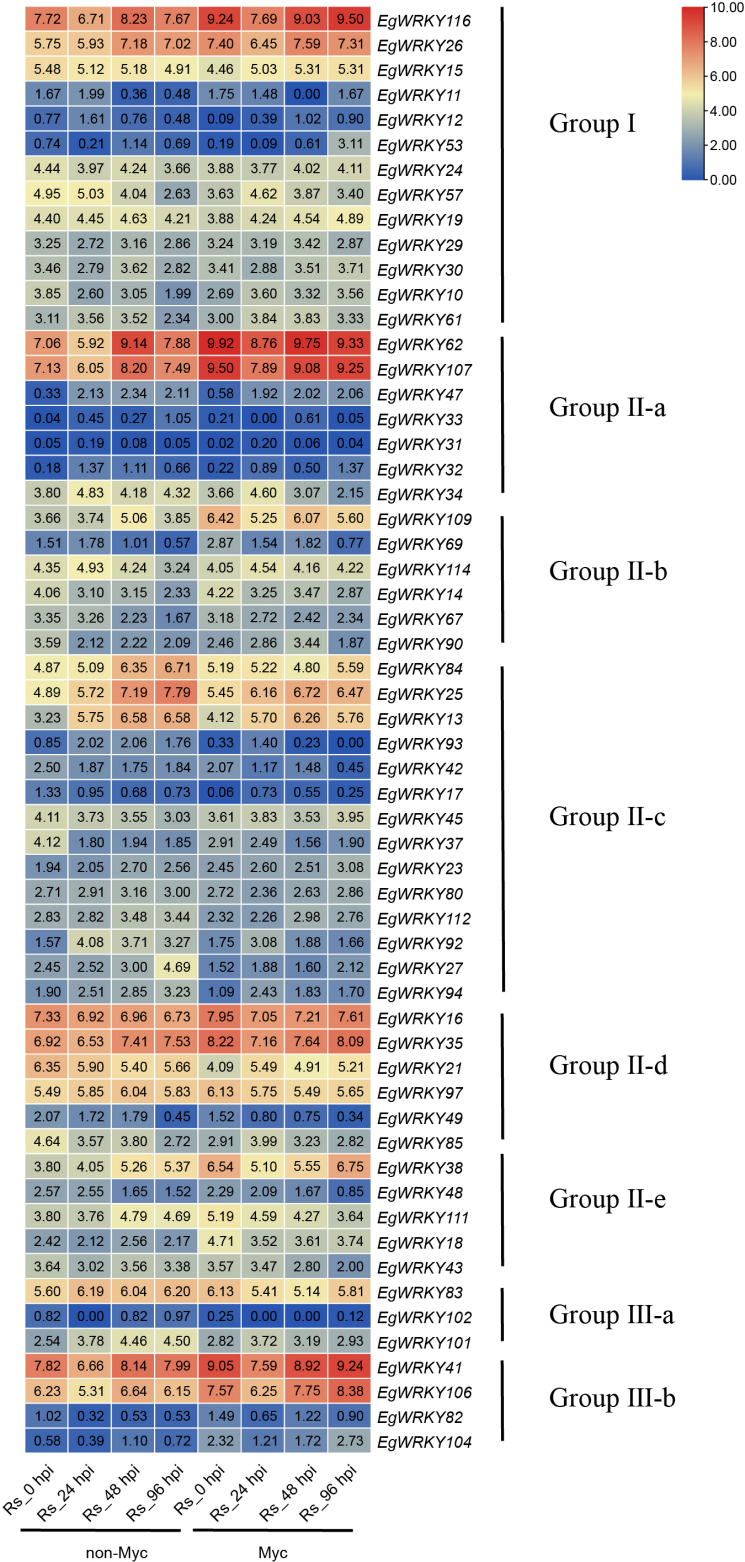
Heatmap of *EgWRKY* gene expression determined by RNA-seq. Log2-fold differences in gene expression (FPKM values) were used to create the heatmap. Myc, mycorrhized with *R. irregularis*; *Rs*, Infected with *R. solanacearum*.

In addition, we identified *cis*-acting elements associated with mycorrhizal symbiosis in the *EgWRKYs*, such as P1BS (GNATATNC), CTTC-motif (NTTCTTGTTN), and AW-box (CG(N)7CNANG) (Shi et al., 2023; Duan et al., 2024). Members of all subfamilies except groups IV-b and IV-c contain AW-boxes. In addition more than 20% of members of groups II-a and II-c contain P1BS, CTTC-motif, and AW-boxes, suggesting that these two subfamilies may have a role in the AMF symbiosis. The W-box has been implicated in plant resistance to *Rs* (Dang et al., 2014; Phukan et al., 2016; Mukhi et al., 2021; Yang et al., 2021). A relatively high number of family members contain W-box elements (71%, 83%, 90%, 85%, 50%, and 67% of group I, II-a, II-b, II-c, II-e, and IV-a members contain W-box elements, respectively, and in other subfamilies, 100% of members contain W-boxes), suggesting that *EgWRKYs* play important roles in the *E. grandis*–AMF–*R. solanacearum* system (**Figure 6B**). In addition, 7 *EgWRKY* genes that exhibited significant differences in expression at different time points between mycorrhizal and non-mycorrhizal *E. grandis* contain W-boxes (**Figures 6C**, **7**).

### 3.6 AMF *R. irregularis* mediates the expression of *EgWRKYs* in response to *R. solanacearum* infection

During the *Rs* infection process, *EgWRKY* genes showed different expression patterns in the roots of *Ri* mycorrhizal and non-mycorrhizal *E. grandis* plants (**Figure 7**). Among the 58 *EgWRKYs*, 21 genes responded to *R. irregularis* in the absence of *Rs* infection. In detail, eight genes were downregulated in response to this treatment, including three from group I (*EgWRKY10*, *EgWRKY15*, and *EgWRKY57*) and five from group II (*EgWRKY90*, *EgWRKY17*, *EgWRKY37*, *EgWRKY21*, and *EgWRKY85*). The remaining 13 *EgWRKYs* were upregulated by this treatment, including two from group I (*EgWRKY26* and *EgWRKY116*), eight from group II (*EgWRKY18*, *EgWRKY35*, *EgWRKY38*, *EgWRKY62*, *EgWRKY69*, *EgWRKY107*, *EgWRKY109*, and *EgWRKY111*), and three from group III-b (*EgWRKY41*, *EgWRKY106*, and *EgWRKY104*). Notably, *EgWRKY116*, *EgWRKY62*, *EgWRKY107*, *EgWRKY109*, *EgWRKY38*, *EgWRKY41*, and *EgWRKY106* were expressed at higher levels in mycorrhizal plants compared to non-mycorrhizal plants. This trend was observed not only in the absence of the pathogen (at 0 h of *Rs* infection), but also at other time points of the pathogenic interaction. These results suggest that the expression of these seven genes is influenced by AMF symbiosis and might enhance *E. grandis* resistance against *Rs*.

Additionally, during *Rs* infection, *EgWRKY116*, *EgWRKY62*, *EgWRKY107*, *EgWRKY41*, and *EgWRKY106* showed a significant decrease in expression at 24 hpi, regardless of mycorrhization, followed by an increase at 48–96 hpi; this trend was observed for *EgWRKY26* only in the mycorrhizal roots of *E. grandis*. In the absence of mycorrhizal treatment, *EgWRKY*57 and *EgWRKY114* showed slowly upregulated expression before 24 hpi followed by downregulation at 48 hpi and reached the lowest expression level at 96 hpi. *EgWRKY*27 expression increased after infection with *Rs* and was quickly and significantly upregulated at 96 hpi. *EgWRKY109* was quickly upregulated at 48 hpi with *Rs*, and at 96 hpi, its expression level returned to that at 24 hpi. These results indicate that these *EgWRKYs* have different expression patterns following infection by *Rs*. *EgWRKY47*, *EgWRKY84*, *EgWRKY13*, and *EgWRKY25* generally showed sustained increases in expression during *Rs* infection. Additionally, the expression of *EgWRKY37*, *EgWRKY42*, and *EgWRKY90* significantly decreased after *Rs* infection compared to the uninfected controls.

## 4 Discussion

WRKY TFs, which play crucial roles in plant responses to abiotic and biotic stress, constitute one of the largest and most important TF families (Rushton et al., 2010; Javed et al., 2022). While the WRKY family has been extensively studied in model plants, the characterization of the WRKY family in the *E. grandi*s–AMF–*R. solanacearum* tripartite interaction is missing. The first version of the *E. grandis* genome contained 79 *WRKY* genes; the responses of these genes to plant hormonal and abiotic stress treatments were previously studied (Fan et al., 2018). However, in the present study, based on the *E. grandis* genome version released in 2021 (ASM1654582v1), we identified 117 *EgWRKYs*, representing an increase of 38 genes in the genome compared to the previous report (Fan et al., 2018). Furthermore, our analysis focused on *EgWRKY* gene expression in the *E. grandis*–*R. irregularis* interaction. Our findings provide insight into *EgWRKY* genes in the context of symbiotic interactions and biotic stress responses.

The prevalence of mutations and deletions in plant WRKY domains has been documented in various studies (Yao et al., 2015; Jiang et al., 2017; Song et al., 2018; Chen et al., 2019). In sunflower (*Helianthus annuus*), five WRKY proteins were found to lack one WRKY structural domain (Li et al., 2020). In maize (*Zea mays*), nine WRKY proteins lack one WRKY structural domain, seven lack WRKY zinc fingers, and three lack one WRKY motif (Hu et al., 2021). The WRKY motif of WRKY genes in eggplant (*Solanum melongena*) was also reported to be mutated (Yang et al., 2020). In *C. annuum*, due to the replacement of Q by M in the conserved motif WRKYGQK, CaWRKY27b in the nucleus failed to bind to W-boxes in the promoters of immunity- and thermotolerance-related marker genes (Yang et al., 2022). In the current study, 21 *EgWRKYs* were found to harbor mutations in WRKY structural domains, suggesting that different *EgWRKYs* may confer different resistance responses or susceptibility traits. Furthermore, the zinc finger at the C-terminus of EgWRKY3, classified as III-a, was found to be mutated to H-X_1_-S, while the zinc finger at the C-terminus of EgWRKY7 was mutated from H-X_1_-C to H-X_2_-C. Lastly, the structure of the zinc finger at the N-terminus of EgWRKY85, classified as II-d, was mutated from C-X_5_-C to C-X_4_-C. These findings suggest that the zinc finger is subject to a substantial number of mutations in group III-a and III-b, potentially leading to the generation of new functions among its members.

The tremendous diversity of WRKY TFs and their unpredictable numbers across plant species might be related to a variety of evolutionary processes, including genome-wide duplications. The success of duplicated genes, whether in tandem or segmental form, during genome-wide duplications could potentially explain ongoing evolutionary shifts and crop domestication (Bertioli et al., 2016; Van De Peer et al., 2017; Bohra et al., 2022). Gene duplication is known to play a major role in the amplification and evolution of plant gene families (Bertioli et al., 2016; Van De Peer et al., 2017). In this study, we observed duplications involving *EgWRKY83*, *EgWRKY103* with *AtWRKY54*, and *EgWRKY106* with *AtWRKY41* (**Figures 4A, B**). Notably, *Rs* specifically targets *AtWRKY54* to suppress plant immune responses to AvrRp4 and PopP2 (Kim et al., 2024). Furthermore, AvrRps4(C) associates with the WRKY domains of the related but distinct RRS1B/RPS4B NLR pair and the DNA-binding domain of AtWRKY41, with similar binding affinities, and effector binding interferes with WRKY-W-box DNA interactions (Mukhi et al., 2021). These results suggest that the orthologs *EgWRKY83*, *EgWRKY103*, and *EgWRKY106* may have similar functions in *E. grandis* during interactions with pathogens, making them interesting targets for further investigation.

While introns are not directly involved in the proteome, they often contain regulatory elements that can modulate protein isoform production, RNA stability, and translational efficiency, thereby amplifying the protein-coding potential of the genome through post-transcriptional splicing of introns (Jacob and Smith, 2017; Keane and Seoighe, 2016; Parenteau et al., 2019; Morgan et al., 2019). Due to their very long introns, *EgWRKY6*, *EgWRKY8*, *EgWRKY9*, and *EgWRKY51* may play important roles in regulating gene transcription and translation; this concept deserves further investigation.

Predictive analysis of the *cis*-acting elements of *EgWRKYs* revealed the presence of many light-associated, plant hormone-associated, tissue-specific, stress-associated, and mycorrhiza-associated elements in the promoters of *EgWRKY* genes (**Figure 6**), which might be involved in a variety of biological processes. In *E. grandis*, more than 50% of the members of subfamilies other than subfamily III-a contain W-box *cis*-acting elements (**Figure 6B**). The W-box flanking regions of its sequence may contribute to the molecular recognition mechanism of *EgWRKY* genes (Cheng et al, 2019; Hsin et al, 2022; Mukhi et al, 2021), which bind to and are activated by other TFs upon the invasion of plants by *Rs* (Mukhi et al., 2021; Phukan et al., 2016; Dang et al., 2014; Yang et al., 2021).

*Eucalyptus* WRKY genes are rich in mycorrhizal-induced *cis*-acting elements such as P1BS, CTTC, and AW-box elements*. EgWRKY41*, which contains both an AW-box and P1BS, was significantly up-regulated in response to AMF inoculation, while *EgWRKY37* and *EgWRKY85* were down-regulated by this treatment, suggesting that different *EgWRKYs* employ different pathways to regulate mycorrhizal interactions in *Eucalyptus*. In *M. truncatula, MtWRKY69* was also shown to be associated with AMF colonization levels (Silvestri et al., 2024). At the transcriptional level, *EgWRKY37*, *EgWRKY84*, *EgWRKY13*, and *EgWRKY25* from the II-c family showed a sustained increase in expression during *Rs* infection, while *EgWRKY37* and *EgWRKY42* showed a significant decrease in expression after *Rs* infection. Therefore, in the *E. grandis*–AMF–*R. solanacearum* interaction system, *EgWRKYs*, encoding core transcription factors, can respond to AMF and *Rs* at the same time. We propose that after mycorrhizal-colonized *E. grandis* is infected by *Rs*, the host reinforces AMF symbiosis by regulating the expression of *EgWRKYs*, thereby enhancing seedling growth while inducing the salicylic acid/jasmonate signaling pathway to enhance host defense against *Rs*.

## 5 Conclusion

We identified 117 *EgWRKY* genes in the *Eucalyptus grandis* genome. The predicted EgWRKY proteins were classified into four groups based on phylogenetic analysis and multiple sequence alignment. Collinearity analysis showed that 25 and 4 of these genes were segmental and tandem duplicates, respectively. All subfamilies contain plant hormone-related *cis*-acting elements (ABREs, TGACG motifs, and CGTCA motifs), which are associated with defense responses. In addition, the majority of subfamilies contain P1BS, CTTC, and AW-box elements; these elements are induced by AMFs. These results suggest that these *EgWRKYs* respond to AMFs and/or *Rs*. We identified 23 and 58 *EgWRKY*s that respond to *R. irregularis* and *R. solanacearum*, respectively, in the *E. grandis*-*Ri*-*Rs* interaction system at the transcriptional level. Several *EgWRKY* genes showed differential expression patterns in mycorrhizal and non-mycorrhizal *E. grandis* roots during *R. solanacearum* infection, and *EgWRKY62*, *EgWRKY107*, and *EgWRKY116* were responsive to the AMF *R. irregularis*. These results provide insights into the characteristics of *E. grandis EgWRKYs* and their potential roles in AMF-mediated defense against *R. solanacearum*.

## Data Availability

The datasets presented in this study can be found in online repositories. The names of the repository/repositories and accession number(s) can be found in the article/**Supplementary Material**.
